# Annual variability of heavy metal content in Svalbard reindeer faeces as a result of dietary preferences

**DOI:** 10.1007/s11356-018-3479-8

**Published:** 2018-10-30

**Authors:** Michał Hubert Węgrzyn, Paulina Wietrzyk, Sara Lehmann-Konera, Stanisław Chmiel, Beata Cykowska-Marzencka, Żaneta Polkowska

**Affiliations:** 1 Prof. Z. Czeppe Department of Polar Research and Documentation, Institute of Botany, Jagiellonian University, Gronostajowa 3, 30-387 Cracow, Poland; 2 Department of Analytical Chemistry, Faculty of Chemistry, Gdańsk University of Technology, 11/12 Narutowicza St., 80-233 Gdańsk, Poland; 3 Department of Hydrology and Climatology, Faculty of Earth Sciences and Spatial Management, Maria Curie-Skłodowska University in Lublin, 2cd Kraśnicka St., 20-718 Lublin, Poland; 4 Laboratory of Bryology, W. Szafer Institute of Botany, Polish Academy of Sciences, Lubicz 46, 31-512 Cracow, Poland

**Keywords:** *Rangifer tarandus platyrhynchus*, Contamination, Pollution, Vegetation

## Abstract

During both winter and summer, Svalbard reindeer selectively feed on different types of vegetation that are not only a source of nutritional value, but also a place of heavy metal accumulation. In the present study, the content of cadmium, chromium, copper, iron, lead, nickel, manganese, and zinc in reindeer excrement was measured. The main aims were to determine the seasonal content of several heavy metals in Svalbard reindeer faeces, and to compare their values in terms of dietary preferences during the year. Summer and winter reindeer excrement was gathered along a designated linear transect running through Bolterdalen and the vegetation described on 1 m^2^ plots. All of the analysed heavy metals were detected in the reindeer faeces and this fact seems to be connected with the incomplete content of these elements in an animal’s tissue after forage digestion. Analysis showed differences between summer and winter excrement in terms of concentrations of cadmium, chromium, iron, and nickel, but no differences were found for the other four elements analysed (manganese, lead, zinc, and copper). However, concentrations of heavy metals in faeces are rather low in comparison with both the levels in the vegetation that may be grazed by reindeer and in reindeer tissue.

**Electronic supplementary material:**

The online version of this article (10.1007/s11356-018-3479-8) contains supplementary material, which is available to authorized users.

## Introduction

Disturbances to wildlife caused by human activity and industrial development pressures are an important environmental issue in Arctic ecosystems. Although polar ecosystems experience less direct impact from human activity compared with other areas, all their abiotic and biotic components receive contaminants due to the long-distance transport of toxic substances from lower latitudes (e.g. Weinbruch et al. 2012). Anthropogenic pollutants, including heavy metals, may accumulate in ecosystems, both directly via atmospheric deposition (Nriagu 1989, 1979) and indirectly through the influence of windblown dust, marine aerosols, and water from melting snow and glaciers (Drbal et al. 1992; Steinnes 1995; Aastrup et al. 2000; Samecka-Cymerman et al. 2011; Ruman et al. 2012; Weinbruch et al. 2012).

One of the priority issues of the Arctic Monitoring Assessment Programme is to monitor and assess heavy metals. According to AMAP (2005), most heavy metals are released into the environment from anthropogenic sources, while Mn and Cr mostly originate from natural sources. Regardless of the origin of heavy metals, they may have a crucial impact on the Arctic environment due to their toxic effects on living organisms. In Arctic terrestrial ecosystems, the majority of heavy metals accumulate in vegetation: vascular plants (Jóźwik 2000), bryophytes (Samecka-Cymerman et al. 2011) and lichens (Garty 2001; Węgrzyn et al. 2013, 2016; Wojtuń et al. 2013), which are also foraged by Svalbard reindeer (*Rangifer tarandus platyrhynchus*). The AMAP report (2005) classified heavy metals into elements that are biologically essential (e.g. Cr, Cu, Ni, Fe, Mn, Zn) or non-essential (e.g. Cd, Pb) for a living organism. According to Clarkson (1986), the first group of elements is toxic only in excess and organisms have developed mechanisms for regulating their levels. The metals in the second group are more likely to have toxic effects on an organism than biologically essential metals; for example, the tolerable content in air-dried forage for domestic animals was estimated to be 500–1000 mg kg^˗1^ for Zn, 0.5 mg kg^˗1^ for Cd, 25–80 mg kg^˗1^ for Cu and 30 mg kg^˗1^ for Pb (National Research Council 1980; Stoltz and Greger 2002).

According to Environmental Monitoring of Svalbard and Jan Mayer (http://www.mosj.no), the total population of Svalbard reindeer is extremely difficult to determine, but in 2015, the local population was estimated to be as high as 1315 in the valley of Adventdalen alone. Furthermore, it was reported that in 2016, a total of 235 reindeer were shot, representing only c. 2.5–5% of the Svalbard population, and therefore did not result in any significant decline. The raw materials obtained from reindeer primarily serve the needs of human consumption; consequently, those who eat reindeer meat can be considered another link in the trophic chain, as they are exposed to the accumulation of anthropogenic contaminants in their tissue.

The accumulation of contaminants in vegetation, and later in animals (herbivores and carnivores), contributes to the circulation of harmful elements and compounds in Arctic terrestrial ecosystems (Hallanger et al. 2011; Węgrzyn et al. 2016). Trophic chains in polar areas consist of a relatively small number of species that are associated with a range of different interactions. Thus, the presence of pollutants in the Arctic environment may even lead to ecological imbalance in the ecosystem (Koivurova 2005). According to Simões and Zagorodnov (2001), Svalbard is one of the areas in the Arctic most affected by anthropogenic pollution due to atmospheric circulation.

The Svalbard reindeer subspecies that inhabits the archipelago is the only ruminant that grazes the tundra vegetation all year round. Because of their high population, which is due to a lack of natural enemies, reindeer occupy all areas of Svalbard not covered by glaciers. The local reindeer populations are considered non-migratory, as a result of which they consume vegetation within a relatively small area (Bjørkvoll et al. 2009; Hansen et al. 2010). During the short, three-month vegetation period, Svalbard reindeer have to eat enough forage to survive and build additional reserves of energy for the winter season (Bjørkvoll et al. 2009; Reimers 1982; Tyler 1986). However, the long winter usually results in the premature depletion of body reserves and a constant search for food under the snow layer (Bjørkvoll et al. 2009; Tyler 1986). Because of specific climate conditions of the archipelago, the diet of Svalbard reindeer is divided into a summer diet, dominated by the green parts and flowers of vascular plants and graminoids, and a winter diet, consisting of cryptogams and woody parts of dwarf-shrubs, which are the only source of food available to reindeer after removing the snow cover (Bjørkvoll et al. 2009; Ekern and Kildemo 1987; Staaland et al. 1993; Węgrzyn et al. 2013). Due to its specific composition of long-living vegetation, the winter diet seems to consist of vegetation characterised by an extended period of heavy metal accumulation (Austrheim et al. 2005; Sancho et al. 2007), the lichens, bryophytes and woody parts of vegetation being exposed to heavy metal accumulation over many years due to fact that they are not annual vegetation. It is therefore assumed that winter forage significantly contributes to the elemental content of reindeer.

The main aim of the present study was to determine the seasonal differences in the contents of selected heavy metals measured in Svalbard reindeer faeces, and to compare these values between the summer and winter periods in reference to reindeer dietary preferences. We set a hypothesis that assumed that contents of heavy metals in summer excrement will be lower than those in winter excrement.

## Material and methods

### Study area and data sampling

**Fig. 1:**
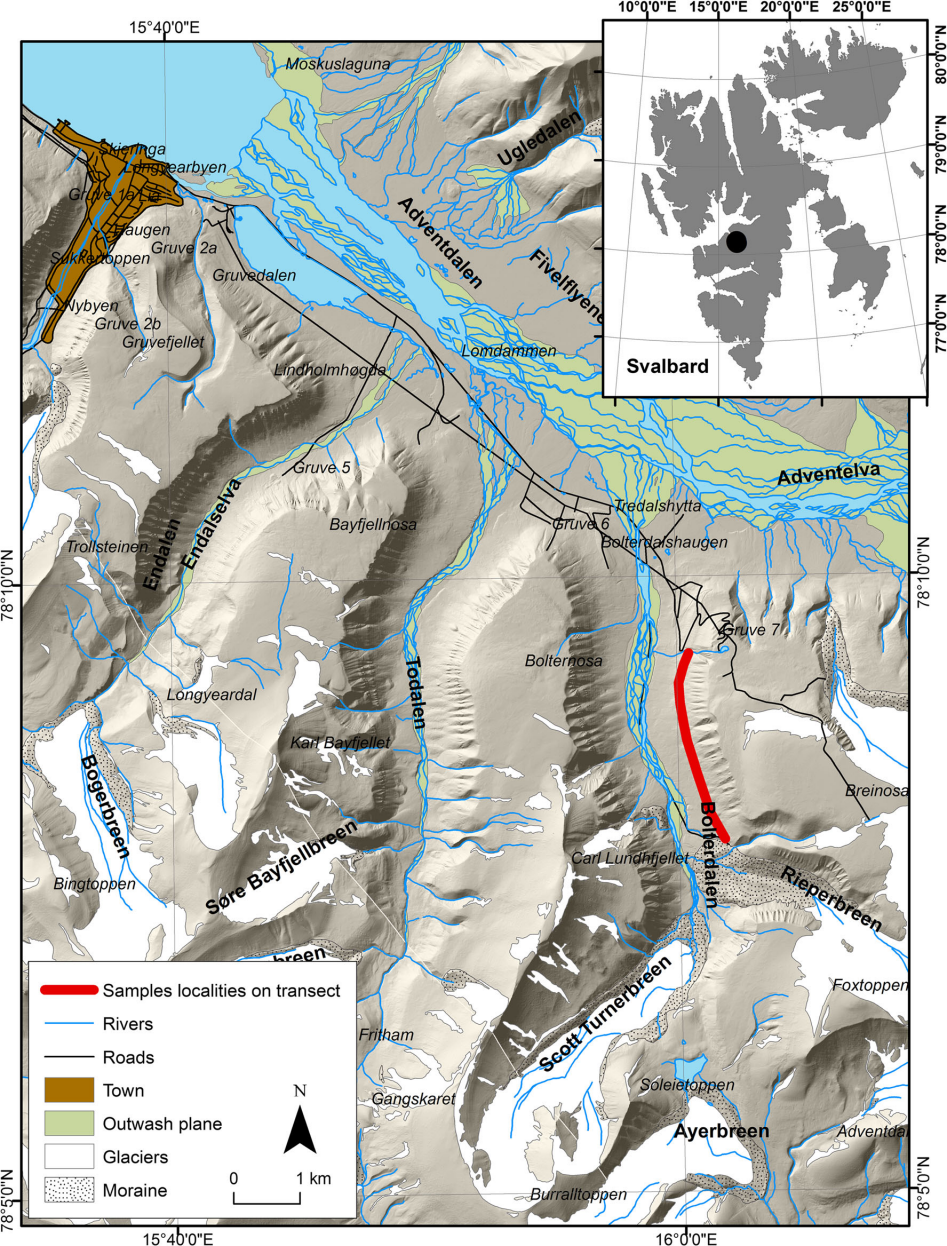
Location of the study area (© Norwegian Polar Institute 2016, www.npolar.no)

Fieldwork was carried out in the summer season of 2015 in Bolterdalen, a glacial valley located in Nordenskiöld Land in central Spitsbergen (Fig. 1) c. 6 km in length and covering c. 7.5 km^2^. The valley is bordered by Bolternosa and Breinosa and enclosed by the Scott-Turnerbreen and Rieperbreen glaciers (Lyså and Lnne 2001).

Summer and winter reindeer excrement was gathered along a linear transect delimited along Bolterdalen (Figs. 1, S1) since a possible difference in shape and consistency between summer and winter excrement was noticed. For summer faeces, only fresh, moist samples were collected, and then dried. Winter faeces were characteristic pellet-shaped droppings and their age for 2014/2015 seasons was determined on the basis of their compact structures, namely no clear cavities as a result of ageing, no cover of cyanobacteria layer on their surface, their black or black-grey surfaces being shiny with no clear matt coating on faeces older than last winter. Both types of excrement were collected at the same 15 sites (30 samples) located between 150 and 250 m a.s.l. The samples were dried, packed in sealed plastic bags and transported to Poland for laboratory analyses.

To identify vegetation from the study area, 1-m^2^ plots were designated at ten selected excrement collection sites along the transect. Within these plots, the percentage cover of vascular plants, bryophytes and lichens was determined according to the scale of Braun-Blanquet (1964).

### Laboratory analyses

Samples of reindeer excrement were lyophilised at − 80 °C and subsequently homogenised and sieved with a 2-mm plastic sieve. Thereafter, 0.5 g of excrement samples was mineralised and extracted in 8 ml of 65% HNO_3_ (suprapur) acid using the Microwave Digestion System (Multiwave GO of Anton Paar). The mineralisation temperature programme was as follows: a 20-min increase in temperature to 180 °C, a 10-min hold in temperature at 180 °C and 10 min of cooling to 60 °C. Following the mineralisation process the samples were transferred quantitatively to 25-ml flasks and filled with dionised water.

Various groups of analyses involved the application of Mili-Q demineralised water (Mili-Q® Ultrapure Water Purification Systems, Millipore® production). Metals (cadmium, chromium, copper, iron, lead, nickel, manganese and zinc) were determined by means of ion coupled plasma mass spectrometry (Thermo Scientific XSERIES 2 ICP-MS) (Table 1). These were analysed using Standard Reference Material NIST 1643e Trace Elements in Water, and Reference Material EnviroMAT ES-L-2 CRM, ES-H-2 CRM SCP SCIENCE. Calibration of the apparatus was based on reference materials supplied by Inorganic Ventures: ANALITYK-CCS-4, CCS-6, CCS-1 and IV-ICPMS-71A.

**Table 1:** Validation parameters and technical specifications used to determine metals

Parameter		Measurement range	LOD^a^	LOQ^b^	Measurement instrumentation
Heavy metals [μg/L]	Cd	0.01–1000	0.01	0.03	Thermo Scientific XSERIES 2 ICP-MS Germany, collision cell technology, cool gas flow Ar: 12 l/min, cell gas flow He/H: 5,5 ml/min
	Cr, Mn, Ni, Cu, Zn, Pb	0.1–1000	0.10	0.30	
	Fe	1.0–1000	1.00	3.00	

^a^The limit of detection (LOD) was calculated on the basis of the standard deviation of the response (s) and the slope of the calibration curve (b), according to the following formula: LOD = 3.3(s/b)

^b^The limit of quantitation (LOQ) was calculated on the basis of the standard deviation of the response (s) and the slope of the calibration curve (b) according to the following formula: LOQ = 10(s/b)

### Statistical analyses

Levene’s test was performed to assess the equality of variances, while the Shapiro–Wilk test helped determine the normality of the data set. The U Mann–Whitney test was used to assess differences in heavy metal concentrations between summer and winter reindeer excrement along the transect.

## Results

### A description of the vegetation in the study area

A general characterisation was prepared of the vegetation along the designated transect as shown in Table S1 which lists the recorded species together with the degree of cover and the phytosociological constancy of each taxon. The vegetation is represented by mesic tundra, dominated by vascular plants (59%), mainly dwarf shrubs, such as *Dryas octopetala*, *Cassiope tetragona* and *Salix polaris*; bryophytes and lichens respectively represented 16% and 20% of the cover.

In the case of bryophytes, *Sanionia uncinata*, *Aulacomnium turgidum* and *Ditrichum flexicaule* were present in all the plots and provided the most cover. Crustose and foliose lichens species were dominant, with *Peltigera venosa*, *Collema ceraniscum* and *Ochrolechia frigida* providing the main components of the biological soil crusts (BSCs). Fruticose lichens were infrequently observed, such as *Flavocetraria nivalis*, a species frequently grazed by reindeer (Ziaja et al. 2016; Olech et al. 2011), but *Stereocaulon alpinum*, *Cetrariella delisei* and *Cladonia macroceras* provided relatively high cover values in all the plots (Table S1).

### Heavy metal content in reindeer excrement

**Fig. 2:**
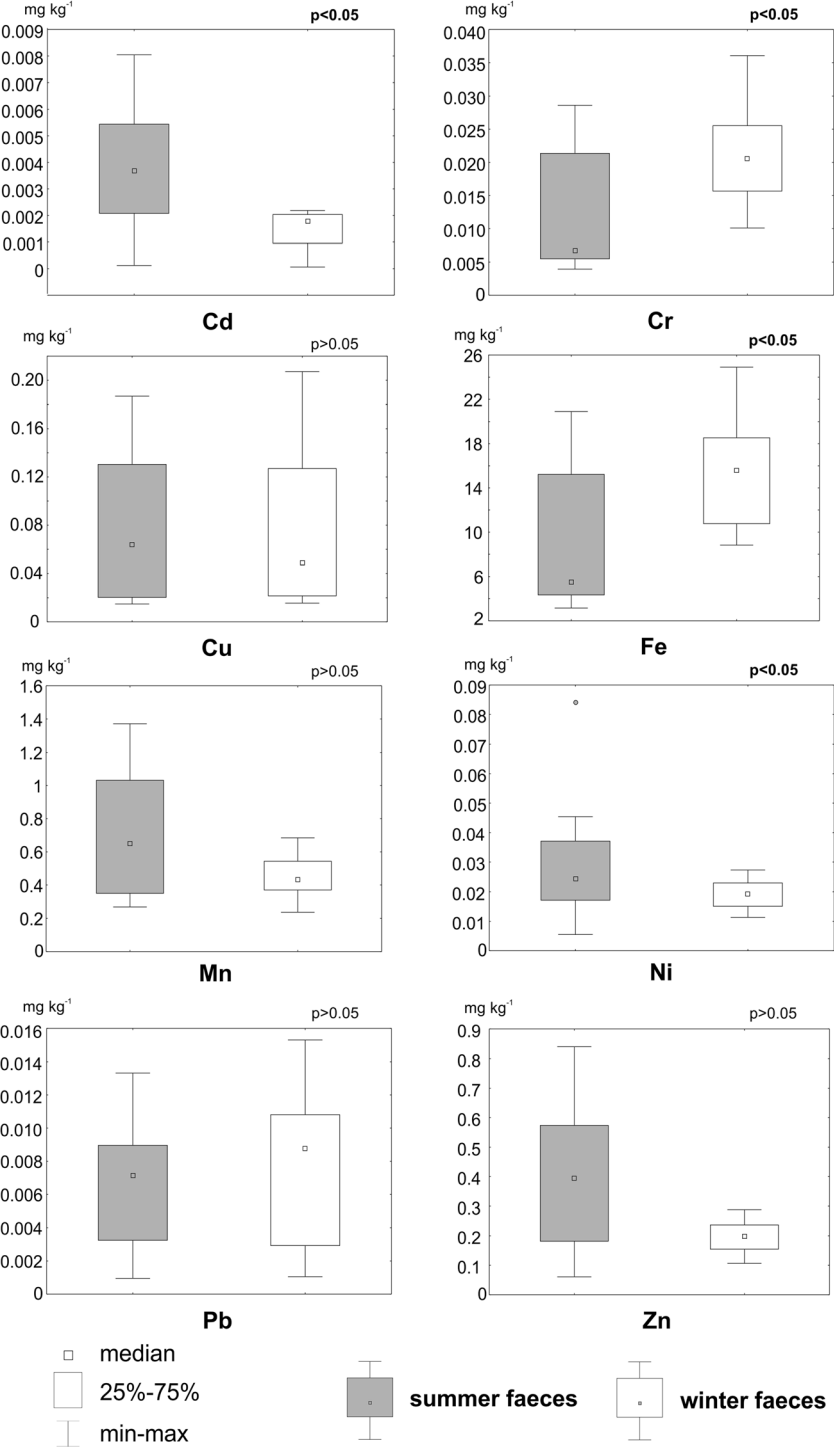
The U Mann–Whitney test comparing heavy metal content between summer and winter excrement. Significant differences (*p* < 0.05) are marked by *p* values highlighted in bold

Eight of the selected heavy metals were detected in the collected reindeer faeces (Table 2, S2); a comparison of between their summer and winter elemental levels is provided in Fig. 2, significant differences confirmed by the U Mann–Whitney test as follows: Cd (U = 39; *p* = 0.002), Cr (U = 57; *p* = 0.022), Fe (U = 56; *p* = 0.02) and Ni (U = 64; *p* = 0.046); however, there were no differences in element contents between summer and winter faeces for the following: Cu (U = 111; *p* = 0.97), Mn (U = 79; *p* = 0.17), Pb (U = 83; *p* = 0.23) and Zn (U = 65; *p* = 0.051).

**Table 2:** The average, standard deviation, minimum and maximum values of heavy metal content [mg kg^−1^] in summer and winter faeces

Element	N	Summer faeces		Winter faeces	
		Average	SD	Average	SD
Cr	15	0.012	0.009	0.02	0.006
Mn	15	0.698	0.378	0.454	0.125
Fe	15	9.51	6.15	14.9	4.52
Ni	15	0.029	0.018	0.018	0.004
Cu	15	0.074	0.061	0.072	0.064
Zn	15	0.371	0.225	0.198	0.056
Cd	15	0.003	0.002	0.001	0.001
Pb	15	0.006	0.003	0.008	0.004

## Discussion

Only a few studies have focused on reindeer excrement as a subject of research (Na et al. 2015; Wang et al. 2015; Zhang et al. 2014; Zhu et al. 2015), all of which examined the presence of organic pollutants known for their carcinogenic and mutagenic properties (e.g. polychlorinated biphenyls, polybrominated biphenyl ethers, polycyclic aromatic hydrocarbons). The literature includes much less information on the metal content of reindeer kidneys (Borch-Iohnsen et al. 1996; Larter and Nagy 2000), livers (Aastrup et al. 2000; Borch-Iohnsen et al. 1996) or muscles (Aastrup et al. 2000). In the case of Svalbard, only Staaland et al. (1983) conducted research on the content of iron, copper, manganese, zinc, molybdenum and cobalt in reindeer excrement, but these samples were gathered directly from distal colons. Because Svalbard reindeer are considered to be non-migratory (Bjørkvoll et al. 2009; Hansen et al. 2010; Tyler and Øritsland 1989), data on the heavy metal content of their faeces may be used for non-invasive contamination monitoring purposes on a local scale.

The presence of all studied heavy metals in reindeer faeces is possibly connected with the incomplete content of these elements in animal tissue after forage digestion as they are excreted in faeces. Nevertheless, the concentrations of heavy metals in the faeces were low compared with those in vascular plants, bryophytes and lichens grazed by the reindeer, and their content in reindeer tissue (Fig. 2; Table 3).

**Table 3:** A comparison of the minimum and maximum content of heavy metals in lichens, bryophytes, vascular plants (Wojtuń et al. 2013), the liver, kidney of Svalbard reindeer (Borch-Iohnsen et al. 1996) together with the obtained element contents in all reindeer excrement. Also the average element contents in faecal samples from distal colon of Svalbard reindeer are given (Staaland et al. 1983)

Heavy metal		Minimum and maximum element content [mg kg^−1^]						Average element content [mg kg^−1^]	
		Lichens	Bryophytes	Vascular plants	Liver	Kidney	Reindeer faeces	Distal colon	
Cd	Min	0.06	0.15	0.29	0.20	0.50	0.001		–
	Max	0.10	0.51	1.08	2.40	23.0	0.008		–
Cr	Min	0.27	2.28	0.38	–	–	0.004		–
	Max	0.83	8.49	1.12	–	–	0.036		–
Cu	Min	0.77	4.85	2.20	3.20	2.20	0.015	Summer	26
	Max	1.33	13.50	6.00	260	7.60	0.207	Winter	11.1
Fe	Min	357	1,97	120	–	–	3.15	Summer	4.39
	Max	629	7,11	353	–	–	24.9	Winter	15.69
Mn	Min	12.0	77.38	47.6	1.20	0.40	0.237	Summer	623
	Max	18.3	298	121	6.50	2.50	1.37	Winter	267
Ni	Min	0.47	1.74	0.60	–	–	0.005		–
	Max	0.90	6.89	2.14	–	–	0.084		–
Pb	Min	1.77	1.49	0.48	0.50	0.60	0.001		–
	Max	2.53	9.95	1.38	6.10	15.0	0.015		–
Zn	Min	11.0	19.5	34.20	15.0	15.0	0.061	Summer	545
	Max	16.3	33.4	65.60	140	38.0	0.839	Winter	148

Although the statistical analysis showed significant differences between summer and winter excrement in terms of the concentrations of the selected heavy metals, it is difficult to draw any unequivocal conclusions on this issue since no other data on the content of elements in reindeer excrement in the Arctic region exist. Furthermore, it is worth underlining the fact that, compared to Norwegian reindeer, Svalbard reindeer are a different subspecies which subsist in different habitat conditions that influence the microbial structure of reindeer rumen (Sundset et al. 2007). The only study on the above-mentioned topic was conducted in 1979 in Adventdalen, where six faecal samples were collected from the distal colon of reindeer shot in the field (Staaland et al. 1983) that showed differences between concentrations of Fe, Cu, Mn, and Zn in summer and winter excrement. When we compare this study with our own results, we can observe that the average heavy metal content in faecal samples from 1979 is much higher (Table 3). These concentrations are also much higher when compared with reindeer tissue and examined vegetation (Table 3), and it is difficult to compare these values with the obtained results.

**Fig. 3:**
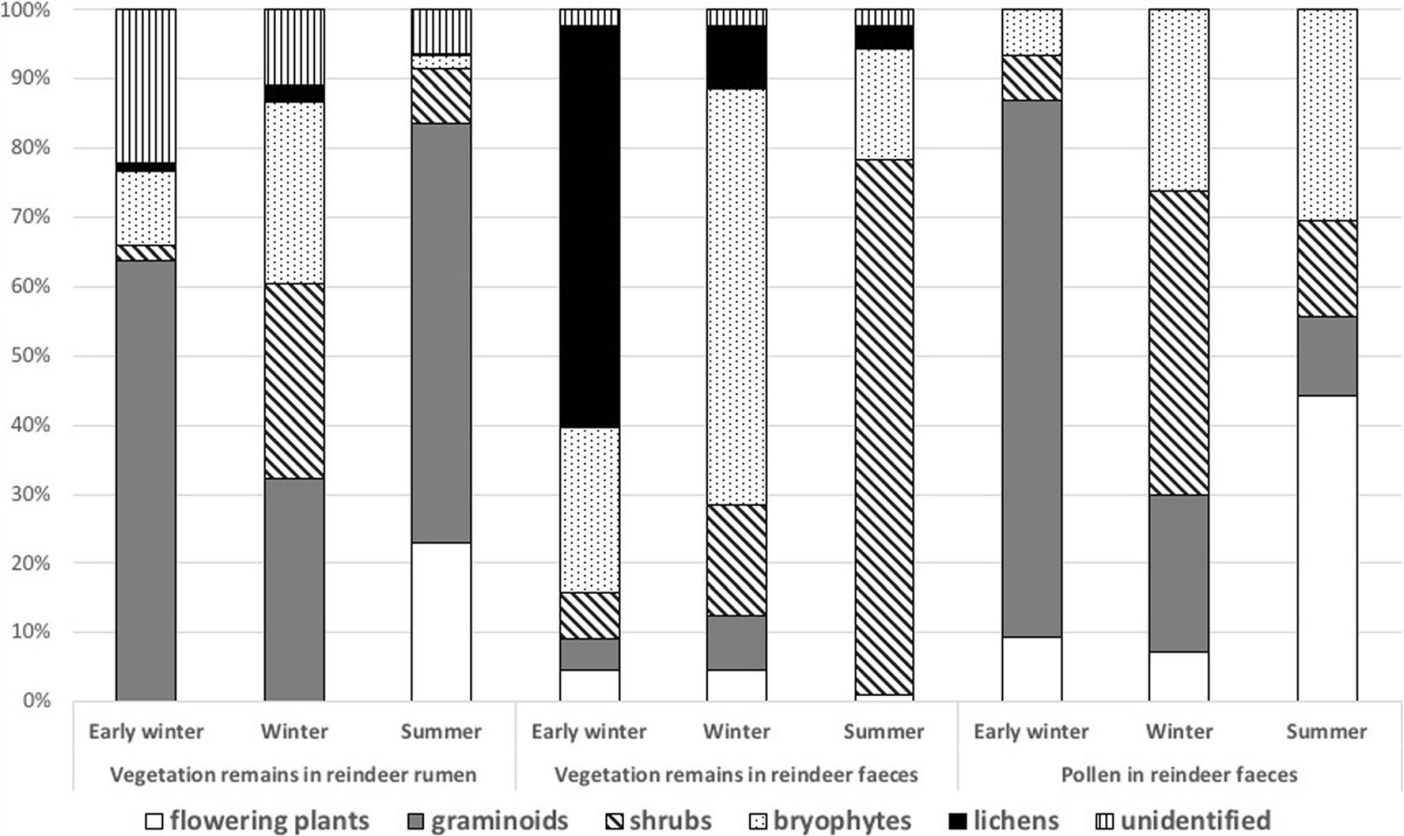
Differences in the percentage composition of reindeer diet in the early winter, winter and summer, based on the species remains investigated in reindeer rumen from a reindeer population in Colesdalen in 1999–2002 (Bjørkvoll et al. 2009) and species remains investigated in reindeer faeces collected in Broggerhalvoya in 1989 (Staaland et al. 1993) and based on the pollen investigated in reindeer faeces collected in Todalen in 1994–1995 (Bjune 2000)

The results can be analysed in terms of reindeer dietary preferences during the Arctic year, the form of available forage probably influencing the heavy metal content in summer and winter faeces (Figs 2 and 3; Table 3). Differences in the percentages of herbaceous plants, shrubs, bryophytes and lichens eaten by reindeer during the summer and winter (Bjørkvoll et al. 2009; Ekern and Kildemo 1987; Staaland et al. 1993), as well as different contents of particular element in such vegetation (Table 2; Wojtuń et al. 2013), are probably a major reason for variations in heavy metal content, both in reindeer tissue (Borch-Iohnsen et al. 1996) and in excrement.

The mesic tundra of the study area is dominated by dwarf shrubs, flowering plants and graminoids, while bryophytes and lichens were less common (Table S1). According to Elvebakk (2005), the vegetation of Bolterdalen is dominated by *Cassiope tetragona*, which is similar to the vegetation of Colesdalen where the research by Bjørkvoll et al. (2009) was undertaken. In the study conducted by Bjune (2000) in Todalen in the valley parallel to Bolterdalen, the characteristic vegetation was similar to that of Bolterdalen and the area investigated by Staaland et al. (1993) in Broggerhalvoya was dominated by *Cassiope tetragona* and *Luzula nivalis* (Elvebakk 2005), which is also similar to that of Bolterdalen. The species composition of mesic tundra has a direct influence on the reindeer’s diet, which in turn dictates the heavy metal content in excrement (Fig. 2; Table 2). Flowering plants and graminoids are the most frequently grazed by reindeer during summer (Fig. 3), but graminoids are also commonly eaten during the early winter (Staaland et al. 1993; Bjune 2000; Bjørkvoll et al. 2009). In contrast, dwarf shrubs, bryophytes and lichens are important components of the reindeer’s winter diet (Staaland et al. 1993; Bjune 2000; Bjørkvoll et al. 2009). In the summertime, reindeer feed on flower petals, while in winter they eat the ligneous parts of plants (Staaland et al. 1993; Bjune 2000; Bjørkvoll et al. 2009).

The obtained results only partly confirmed the hypothesis that assumed that contents of heavy metals in summer excrement will be lower than in winter excrement. Contents of Cd were higher in the summer faeces, which may be connected with the easy accumulation of Cd in the leaves and flowers of vascular plants (Kabata-Pendias 2011). In Svalbard, Wojtuń et al. (2013) claimed that vascular plants accumulated Cd within a range of 0.29–1.08 mg kg^−1^ (Table 3), but in a previous study by Jóźwik (2000), this figure could reach 8 mg kg^−1^. However, a lower Cd content has been observed in lichens, bryophytes, and the ligneous parts of vascular plants (Table 3, Wojtuń et al. 2013), which may explain the differences observed in winter and summer excrement. In comparison to reindeer tissue, the Cd content in vascular plants is rather low (Table 3). However, higher levels of Cd accumulation were observed in reindeer tissue, mainly in the kidneys, which may be the reason for the lower levels of this element in excrement (Fig. 2).

The Cr content was higher in winter excrement. According to Kabata-Pendias (2011), the concentration of Cr is higher in bryophytes than in vascular plants. Reindeer frequently feed on bryophytes during the early winter and winter (Fig. 3), which may directly result in a higher Cr content in winter faeces. Similarly, the Fe content is also higher in winter excrement, and was also observed to be higher in bryophytes than in vascular plants, and indeed in lichens (Table 3). The higher proportion of bryophytes and lichens in winter forage may directly result in a high Fe content in winter excrement. However, data are not available on Fe content in reindeer tissue.

The Mn content seems to be similar in winter and summer excrement (Fig. 2). Mn in vascular plants, the content of which can be metabolically controlled, mainly accumulates in leaves and may reach 121 mg kg^−1^ (Kabata-Pendias 2011). On the other hand, its concentration in bryophytes can be twice as high as in vascular plants (Table 3). The high proportion of forage consisting of vascular plants in summer and bryophytes in winter, together with the accumulation of Mn in the liver and kidney, appear to explain its consistent level in excrement across the different seasons. A slightly higher content of Ni was noted in summer excrement (Fig. 2), which may be due to its high content in the leaves and seeds of graminoids, flowering plants and shrubs (Kabata-Pendias 2011). However, its high content was also noted in bryophytes, which may explain the similar Ni level in the winter excrement.

There was no difference in Pb content between the summer and winter excrement (Fig. 2). This element accumulates similar levels in both vascular plants and cryptogams (Table 3). The high content of Pb in reindeer kidneys and livers might be a factor influencing its content in faeces (Table 3). The difference in Zn content between summer and winter excrement has not been confirmed by statistical analysis (Fig. 2). As with Pb, this element has accumulated at high levels in reindeer livers and kidneys, and this may have an indirect influence on its content in faeces.

Analysing the heavy metal contents in vegetation and in animals fed upon it, the local sources of contamination also play an important role. As regards Borteldalen, situated c. 300 m above the valley, the coal dust which is emitted from the still active Gruve 7 coal mine may affect the content of heavy metals in vegetation grazed by reindeer, and may be deposited on the surface of faeces. The conducted studies (e.g. Pandey et al. 2014) showed that coal dust may influence the content of heavy metals, but in Svalbard there has been no detailed investigation of its deposition and impact on both vegetation and animals. Due to the fact that from the second half of the twentieth century Norwegian coal mines have been successively closed, further comparative analyses of heavy metal content will allow the scale of future pollutant changes to be estimated.

## Conclusions

Despite its preliminary character, the research investigated the heavy metals in reindeer excrement. Differences in the concentration of heavy metals in winter and summer excrement would appear to be as a consequence of the variability of reindeer forage during the year, as well as possibly being influenced by their accumulation in reindeer tissue. The presented study is a starting point for future research on heavy metal contents and non-invasive monitoring of pollutants in Arctic ecosystems.

## Electronic supplementary material

Fig. S1 Svalbard reindeer in Bolterdalen (A) and their winter (B) and summer (C) faeces (PNG 6745 kb)

High Resolution Image (TIF 47104 kb)

Table S1 (DOCX 21.2 kb)

Table S2 (DOCX 18 kb)
