# Supplementary figures and images for: Annual variability of heavy metal content in Svalbard reindeer faeces as a result of dietary preferences
Source: Environ Sci Pollut Res Int. 2018 Oct 30;25(36):36693–701. doi: 10.1007/s11356-018-3479-8 (PMC6290696; doi:10.1007/s11356-018-3479-8)

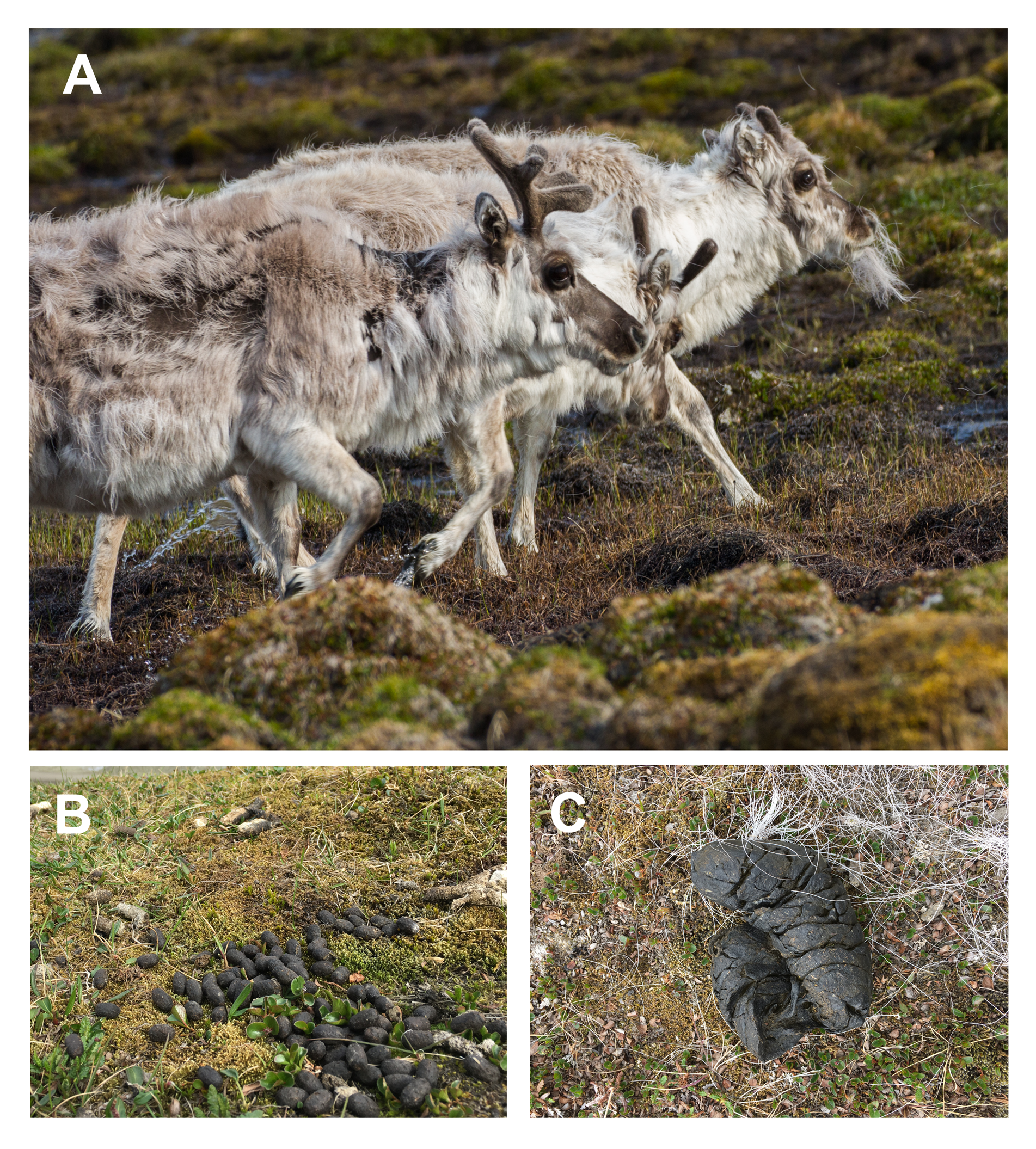

Supplement: Supplementary file 1 — Svalbard reindeer in Bolterdalen (A) and their winter (B) and summer (C) faeces (PNG 6745 kb) [file 11356_2018_3479_Fig4_ESM.png]

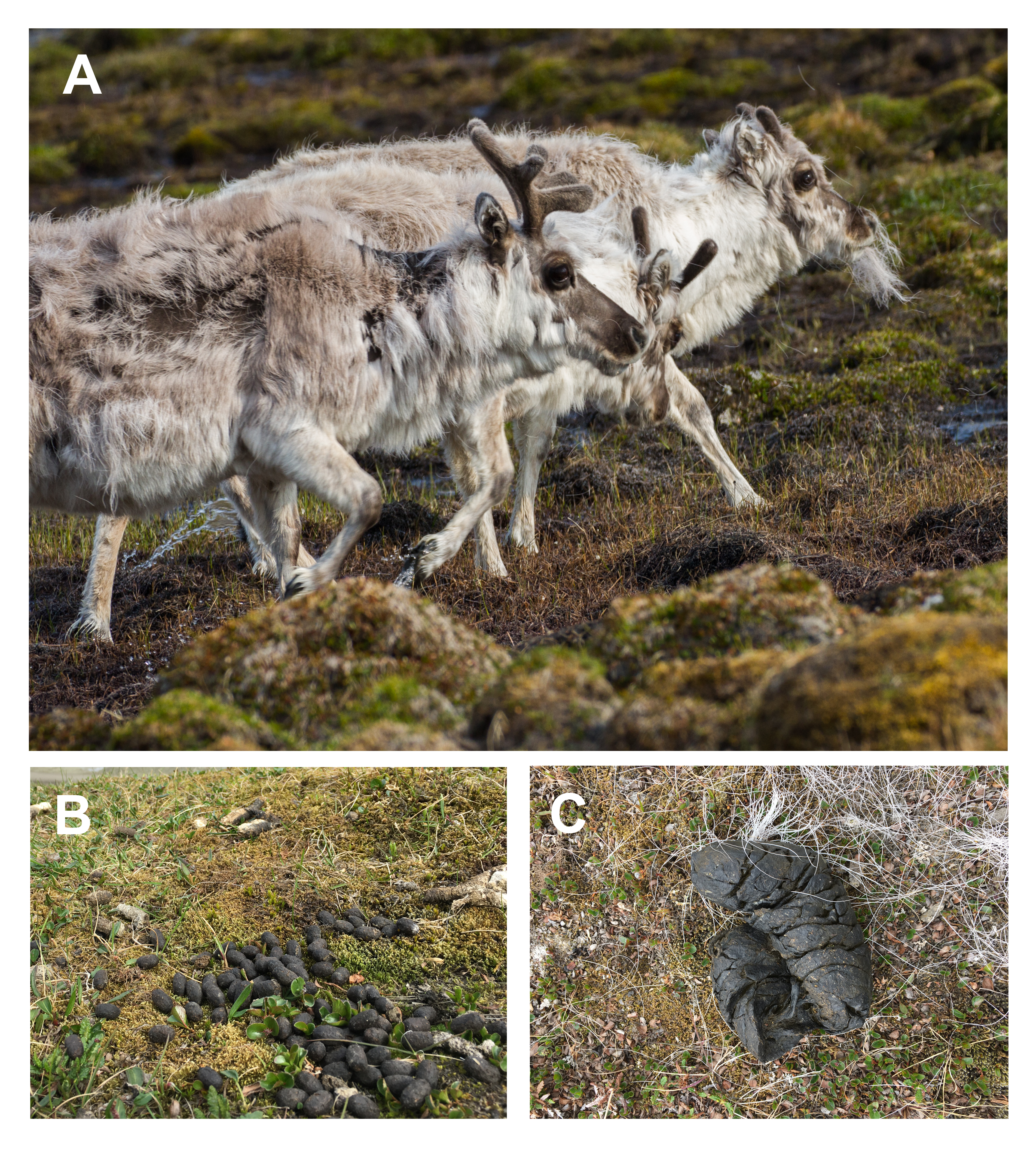

Supplement: Supplementary file 2 — High Resolution Image (TIF 47104 kb) [file 11356_2018_3479_MOESM1_ESM.tif]
