# Supplementary material for: Annual variability of heavy metal content in Svalbard reindeer faeces as a result of dietary preferences
Source: Environ Sci Pollut Res Int. 2018 Oct 30;25(36):36693–701. doi: 10.1007/s11356-018-3479-8 (PMC6290696; doi:10.1007/s11356-018-3479-8)
Supplement: Supplementary file 3 — (DOCX 21.2 kb) [file 11356_2018_3479_MOESM2_ESM.docx]

Table S1 Synthetic table of vegetation showing the species cover according to scale: r — 0.1%; + — 0.5%; 1 — < 5%; 2 — 5% – 25%; 3 — 25% – 50%; 4 — 50% – 75%; 5 — 75% – 100% and stability of each taxon (percentage of sampling sites in which species was recorded): I — 0% – 20%; II — 20% – 40%; III — 40% – 60%; IV — 60% – 80%; V — 80% – 100% (Braun-Blanquet 1964).

| **Species** | **1** | **2** | **3** | **4** | **5** | **6** | **7** | **8** | **9** | **10** | **Stability** |
| --- | --- | --- | --- | --- | --- | --- | --- | --- | --- | --- | --- |
| **Vascular plants** | | | | | | | | | | | |
| *Dryas octopetala* | 3 | 2 | 2 | 3 | 3 | 3 | 3 | 3 | 3 | 3 | V |
| *Salix polaris* | 1 | 3 | 1 | 1 | 3 | 2 | 2 | 2 | 1 | 1 | V |
| *Bistorta vivipara* | + | + | 1 | 2 | 2 | 2 | 1 | + | 2 | 1 | V |
| *Poa alpina* var*. vivipara* | + | 1 | + | + | 2 | 1 | + | + | + | 1 | V |
| *Cassiope tetragona* | 1 | + | + | 1 | 1 | + | 1 | 1 | 1 | + | V |
| *Luzula confusa* | + | + |  | + | 1 | r | + | + | r | + | V |
| *Cerastium arcticum* |  | + | + | 1 | 1 | + |  |  |  | 1 | IV |
| *Cerastium regelii* |  |  | + | + |  | + | + |  | + | 1 | IV |
| **Bryophytes** | | | | | | | | | | | |
| *Sanionia uncinata* | 1 | 1 | 1 | 1 | 1 | 1 | + | + | + | 1 | V |
| *Aulacomnium turgidum* | + | 1 | + | 1 | 1 | 1 | 1 | + | 1 | + | V |
| *Ditrichum flexicaule* | 1 | 1 | 1 | 1 | 1 | + | + | + | + | 1 | V |
| *Dicranum scoparium* | 1 | 1 | 1 | 1 | 1 | + | + | + | + | 1 | V |
| *Tritomaria quinquedentata* | + | + | + | 1 | 1 | 1 | 1 | 1 | 1 | + | V |
| *Scorpidium scorpioides* | 1 | 1 | 1 | 1 | 1 | + | r | + | + | 1 | V |
| *Hygrohypnum alpestre* | r | + | 1 | + | r | + | + | r | r | r | V |
| *Timmia norvegica* | r | + | 1 | + | + | + | r | r | r | r | V |
| *Dicranum laevidens* | r | + | 1 | + | r | + | r | r | r | r | V |
| *Lophozia badensis* | r | + | + | + | r | + | r | r | r | r | V |
| *Racomitrium lanuginosum* | + | + |  | 2 | + | + | 1 | + | 2 | + | V |
| *Syntrichia ruralis* | + | + |  | + | + |  | r | r | + | r | IV |
| *Bartramia ithyphylla* | + | + | r | + | + | r | r |  | + |  | IV |
| **Lichens** | | | | | | | | | | | |
| *Stereocaulon alpinum* | 1 | 2 | 1 | 2 | 1 | 2 | 1 | 1 | 2 | + | V |
| *Collema ceraniscum* | 1 | + | + | 2 | 1 | 2 | 1 | 1 | + | + | V |
| *Ochrolechia frigida* | + | 1 | + | 1 | + | 1 | 1 | 1 | 2 | + | V |
| *Protopannaria pezizoides* | 1 | + | + | + | + | + | + | 1 | 1 | 1 | V |
| *Cladonia pyxidata* | + | + | + | + | + | + | + | 1 | 1 | 1 | V |
| *Cladonia macroceras* | r | r | 1 | 1 | + | + | + | + | 1 | + | V |
| *Cetrariella delisei* | r | + | + | 1 | + | + | + | + | + | + | V |
| *Ochrolechia androgyna* | + | + | r | + | + | + | + | 1 | + | + | V |
| *Peltigera venosa* | 3 | 1 | 2 |  | + | + |  | + | + | + | IV |
| *Peltigera didactyla* |  | 2 |  | + | + | + | 1 | 1 | 1 | + | IV |
| *Cladonia mitis* |  | + | + |  |  | + |  | + | + |  | III |
| *Psoroma hypnorum* | + |  |  | + | + | r |  |  |  | + | III |

**Vascular plants: II** – *Cochlearia groenlandica, Oxyria digyna, Draba alpina;* **I** – *Saxifraga caespitosa, Saxifraga cernua, Saxifraga hieracifolia, Draba lactea, Festuca rubra, Pedicularis dasyantha, Saxifraga oppositifolia;* **Bryophytes: I –** *Ptilidium ciliare, Racomitrium ericoides, Barbilophozia kunzeana, Blepharostoma trichophyllum;* **Lichens: II –** *Peltigera leucophlebia, Cladonia coccifera, Flavocetraria cucullata,* **I –** *Flavocetraria nivalis, Caloplaca tiroliensis, Caloplaca ammniospila, Solorina crocea, Biatora subduplex, Lecidea ementiens, Arthrorhaphis alpina, Polyblastia schereriana, Rinodina turfacea.*
