# Supplementary material for: Annual variability of heavy metal content in Svalbard reindeer faeces as a result of dietary preferences
Source: Environ Sci Pollut Res Int. 2018 Oct 30;25(36):36693–701. doi: 10.1007/s11356-018-3479-8 (PMC6290696; doi:10.1007/s11356-018-3479-8)
Supplement: Supplementary file 4 — (DOCX 18 kb) [file 11356_2018_3479_MOESM3_ESM.docx]

Table S2. Content of heavy metals measured in each reindeer excrement sample.

| **Sample type** | **Heavy metal contents [mg kg^-1^]** | | | | | | | |
| --- | --- | --- | --- | --- | --- | --- | --- | --- |
|  | **Cd** | **Cr** | **Cu** | **Fe** | **Mn** | **Ni** | **Pb** | **Zn** |
| Summer | 0.0001 | 0.028 | 0.064 | 20.9 | 0.31 | 0.084 | 0.001 | 0.061 |
| Summer | 0.002 | 0.02 | 0.018 | 16.9 | 0.65 | 0.021 | 0.003 | 0.19 |
| Summer | 0.008 | 0.006 | 0.19 | 4.8 | 1.2 | 0.041 | 0.009 | 0.84 |
| Summer | 0.003 | 0.005 | 0.089 | 4.5 | 0.27 | 0.006 | 0.004 | 0.36 |
| Summer | 0.002 | 0.014 | 0.066 | 10.3 | 0.75 | 0.017 | 0.008 | 0.39 |
| Summer | 0.002 | 0.021 | 0.078 | 13.9 | 0.47 | 0.017 | 0.013 | 0.19 |
| Summer | 0.004 | 0.029 | 0.034 | 18.6 | 0.35 | 0.026 | 0.011 | 0.18 |
| Summer | 0.002 | 0.024 | 0.025 | 15.2 | 0.28 | 0.020 | 0.009 | 0.16 |
| Summer | 0.001 | 0.014 | 0.018 | 10.9 | 0.36 | 0.013 | 0.007 | 0.087 |
| Summer | 0.005 | 0.006 | 0.015 | 5.4 | 1.4 | 0.032 | 0.003 | 0.53 |
| Summer | 0.006 | 0.004 | 0.16 | 3.2 | 1.3 | 0.036 | 0.003 | 0.57 |
| Summer | 0.007 | 0.007 | 0.13 | 5.5 | 1.0 | 0.045 | 0.003 | 0.58 |
| Summer | 0.005 | 0.005 | 0.020 | 4.3 | 0.57 | 0.023 | 0.004 | 0.40 |
| Summer | 0.005 | 0.006 | 0.038 | 4.0 | 0.72 | 0.024 | 0.007 | 0.41 |
| Summer | 0.005 | 0.004 | 0.18 | 4.1 | 0.85 | 0.037 | 0.009 | 0.61 |
| Winter | 0.001 | 0.017 | 0.16 | 13.1 | 0.39 | 0.021 | 0.003 | 0.29 |
| Winter | 0.001 | 0.016 | 0.13 | 12.7 | 0.39 | 0.016 | 0.002 | 0.18 |
| Winter | 0.0008 | 0.024 | 0.18 | 18.3 | 0.67 | 0.021 | 0.003 | 0.29 |
| Winter | 0.001 | 0.036 | 0.016 | 24.9 | 0.44 | 0.027 | 0.015 | 0.12 |
| Winter | 0.002 | 0.023 | 0.039 | 16.0 | 0.48 | 0.019 | 0.011 | 0.24 |
| Winter | 0.002 | 0.027 | 0.051 | 18.5 | 0.37 | 0.023 | 0.013 | 0.15 |
| Winter | 0.001 | 0.023 | 0.051 | 15.8 | 0.51 | 0.023 | 0.009 | 0.26 |
| Winter | 0.002 | 0.016 | 0.038 | 10.6 | 0.59 | 0.014 | 0.010 | 0.24 |
| Winter | 0.002 | 0.016 | 0.016 | 10.8 | 0.68 | 0.015 | 0.012 | 0.21 |
| Winter | 0.001 | 0.021 | 0.022 | 15.6 | 0.24 | 0.015 | 0.009 | 0.11 |
| Winter | 0.002 | 0.016 | 0.017 | 11.5 | 0.33 | 0.018 | 0.01 | 0.15 |
| Winter | 0.002 | 0.011 | 0.044 | 9.8 | 0.54 | 0.015 | 0.006 | 0.20 |
| Winter | 0.002 | 0.026 | 0.068 | 20.0 | 0.37 | 0.023 | 0.009 | 0.16 |
| Winter | 0.002 | 0.010 | 0.049 | 8.8 | 0.38 | 0.011 | 0.008 | 0.17 |
| Winter | 0.0001 | 0.027 | 0.21 | 18.7 | 0.43 | 0.02 | 0.001 | 0.22 |
